# Supplementary material for: Genome-wide CRISPR Screens in T Helper Cells Reveal Pervasive Crosstalk between Activation and Differentiation
Source: Cell. 2019 Feb 7;176(4):882–896.e18. doi: 10.1016/j.cell.2018.11.044 (PMC6370901; doi:10.1016/j.cell.2018.11.044)
Supplement: Data S2. Processed Data from All the Steps of the Analysis, Related to Figure 1 [file mmc2.zip › supplemental data/motif analysis/Pparg_homer/homerResults/motif7.similar.html]

motif7

## Information for motif7

A
C
G
T
G
A
C
T
A
G
C
T
T
G
A
C
G
C
T
A
A
C
T
G
C
G
A
T
G
C
T
A
A
C
G
T
A
T
C
G
C
G
T
A
T
A
G
C
  
Reverse Opposite:  

A
T
C
G
G
C
A
T
T
A
G
C
T
G
C
A
C
G
A
T
G
C
T
A
T
G
A
C
C
G
A
T
A
C
T
G
T
C
G
A
C
T
G
A
T
G
C
A
  

|  |  |
| --- | --- |
| p-value: | 1e-150 |
| log p-value: | -3.455e+02 |
| Information Content per bp: | 1.700 |
| Number of Target Sequences with motif | 881.0 |
| Percentage of Target Sequences with motif | 7.04% |
| Number of Background Sequences with motif | 920.5 |
| Percentage of Background Sequences with motif | 2.57% |
| Average Position of motif in Targets | 97.0 +/- 54.1bp |
| Average Position of motif in Background | 101.0 +/- 65.8bp |
| Strand Bias (log2 ratio + to - strand density) | 0.1 |
| Multiplicity (# of sites on avg that occur together) | 1.06 |
| Motif File: | file (matrix) reverse opposite |

### Similar de novo motifs found

|  |  |  |  |  |  |  |  |
| --- | --- | --- | --- | --- | --- | --- | --- |
| Rank | Match Score | Redundant Motif | P-value | log P-value | % of Targets | % of Background | Motif file |
| 1 | 0.747 | A C G T A C G T A C G T A G T C T G C A A T G C A C G T G C A T | 1e-113 | -260.453006 | 21.61% | 14.11% | motif file (matrix) |
| 2 | 0.757 | T A C G C T G A C G T A T G C A T A C G A G C T T C A G C G T A T C G A G C T A | 1e-110 | -255.329841 | 12.43% | 6.84% | motif file (matrix) |
| 3 | 0.775 | G A T C C G A T A G T C C G T A A G C T C G A T G T A C G C A T T C A G G C T A | 1e-97 | -225.419787 | 9.66% | 5.05% | motif file (matrix) |
| 4 | 0.703 | C T A G A T C G A C G T C A G T G A C T A T G C G C T A A T G C G A C T A G C T G C A T G A T C | 1e-68 | -157.371437 | 2.20% | 0.62% | motif file (matrix) |
